# Retropharyngeal hematoma secondary to cervical hyperextension in a minor collision trauma presenting with dyspnoea: A case report

**DOI:** 10.1097/MD.0000000000021528

**Published:** 2020-07-31

**Authors:** Jong Hyun Baek, Jung Hee Kim

**Affiliations:** a Department of Thoracic & Cardiovascular Surgery, College of Medicine, Yeungnam University, Daegu; b Department of Thoracic & Cardiovascular Surgery, Korea University Anam Hospital, Seoul, Korea.

**Keywords:** minor trauma, missed injury, retropharyngeal hematoma, risk factors, tertiary survey

## Abstract

**Rationale:**

Massive retropharyngeal hematoma secondary to a minor blunt trauma is rare and easy to be missed in emergency settings due to the absence of visible tissue injury, especially in young patients. However, missing this pathology is dangerous and can result in airway obstruction and even death. Therefore, an effective diagnostic strategy must be developed and thoroughly performed to minimize missed retropharyngeal hematoma.

**Patient concerns:**

A 49-year-old man with a brief cervical hyperextension secondary to a minor collision presented with mild respiratory discomfort. No externally visible injuries were found; however, dyspnea was persistent and aggravating.

**Diagnosis:**

Lateral neck X-ray, neck computed tomography, and spine magnetic resonance imaging revealed a huge retropharyngeal hematoma obstructing the upper airway, without any severe fracture or ligament injury.

**Interventions:**

An emergent orotracheal intubation followed by imaging studies was performed to resolve the aggravating dyspnea. Neck exploration surgery was immediately performed for rapid absorption of the hematoma, bleeding control, and identification of the reason of the pathology.

**Outcomes:**

The surgery was successful, and the patient was discharged without any postoperative sequelae on the 30^th^ postoperative day.

**Conclusion:**

Retropharyngeal hematoma that develops in young patients without visible injuries or severe symptoms after a minor trauma can easily go undetected. Although most hematomas under observation resolve spontaneously, the retropharyngeal hematomas missed at initial assessment may result in critical complications. High level of suspicion, repeated neck CT, and thorough tertiary survey in emergency rooms are helpful in avoiding missed life-threatening retropharyngeal hematoma.

## 1 Introduction

Retropharyngeal hematoma by minor trauma is extremely rare and difficult to identify but potentially fatal.^[1–4]^ Missed retropharyngeal hematoma is critical because this space-occupying lesion may be a cause of airway obstruction due to its location, leading to death even after minor trauma.

Retropharyngeal hematoma after minor trauma is easy to be missed. In addition to spontaneous development, various other reasons that can cause retropharyngeal hematoma include anticoagulant therapy, infection, foreign body ingestion, vascular lesion, and trauma.^[1–3]^ Interestingly, major trauma is usually associated with retropharyngeal hematoma, although minor trauma may also cause huge hematoma and is usually associated with delayed retropharyngeal hematoma.^[1,4]^ The insidious progression of the hematoma over several hours delays symptoms and causes the hematoma to become huge. Furthermore, 1 study reported a relationship between hyperextension and a huge hematoma.^[5]^

Reducing missed injury and minimizing morbidity and mortality are major issues in trauma patients in the emergency department.^[6]^ Missed injuries may present as critical complications and even mortality. Various researches to reduce missed injuries have been performed. Advanced Trauma Life Support (ATLS), the most widely used diagnostic guidelines, comprises a primary survey to detect the most life-threatening injuries and a secondary survey to identify all injuries.^[7]^ However, they are not sufficient to detect all the injuries, and the incidence rate of missed injuries remains considerable. One literature review study reported the rate of missed injuries as 1.3% to 39%.^[6]^ Another large cohort study of trauma patients reported an incidence rate of missed injuries as 8.2%.^[8]^

Thus, a novel strategy for diagnosis should be considered for missed retropharyngeal pathology associated with minor trauma to prevent delayed, potentially fatal complications. We report a case of a missed injury in a minor collision that presented with a massive life-threatening retropharyngeal hematoma only 2 hours after discharge from another emergency center where physical examination, neck lateral X-ray, and laboratory studies showed no abnormal findings.

## 2 Case report

A 49-year-old man with minor trauma from a small road crash presented to our hospital emergency center 2 hours after discharge from a secondary hospital. He was driving a truck with his seatbelt fastened when he had a minor collision with the car in front, and his neck was momentarily hyperextended. The patient immediately visited a secondary hospital emergency room with mild respiratory discomfort without any external injuries. Physical examination, X-ray, and laboratory studies identified no definite visible pathologic findings, and the patient was discharged from the hospital with a recommendation to revisit the emergency room upon onset of any symptoms. However, respiratory insufficiency persisted, and he presented to our tertiary medical center only 2 hours after his discharge.

The patient was alert and orientated and complained only of respiratory discomfort. There were no abnormal findings in the neurological examination. Physical examination revealed no other pathological findings except a slightly edematous neck. He had neither tenderness nor a mass on his neck on palpation. The patient had a blood pressure of 130/80 mm Hg, pulse of 50 beats/min, a 99% oxygen saturation level, and a respiratory rate of 20 breaths/min, and he appeared in mild distress. Initial laboratory test results were as follows: white blood cell (WBC) count, 13,080/μL; platelet count, 244,000/dL; erythrocyte sedimentation rate (ESR), 2 mm/h; and C-reactive protein (CRP), 17 mg/L. Arterial blood gas values were as follows: pH, 7.440; pO2, 77.7 mm Hg; pCO2, 36.8 mm Hg; and HCO3, 24.4 mEqL^−1^ with oxygen mask. As his dyspnea had aggravated, emergent orotracheal intubation was performed. The endotracheal intubation was difficult due to pharyngeal compression; however, it was successful. The patient's past medical history was unremarkable and did not include use of anticoagulants or anti-platelet agents. Laboratory studies, including coagulation profile and platelet count, were within normal limits.

**Figure 1:**
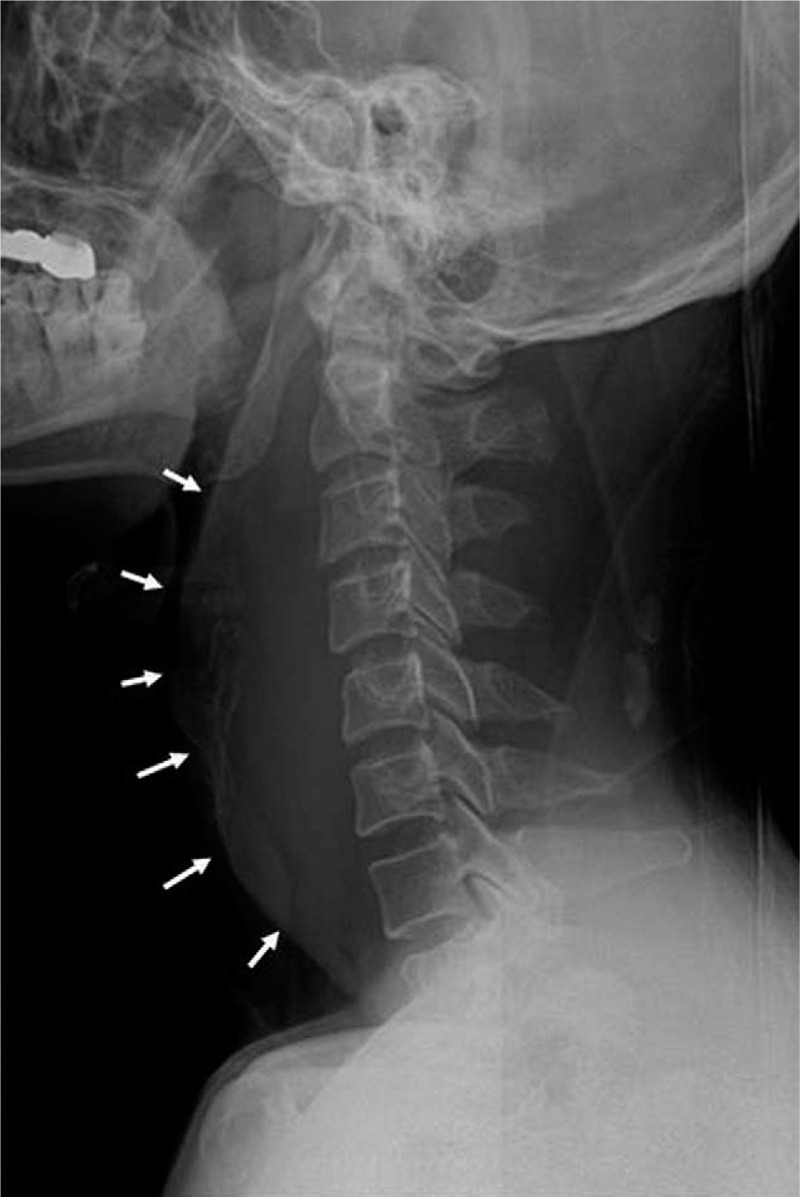
Initial lateral neck X-ray shows marked widening of prevertebral space (arrows).

**Figure 2:**
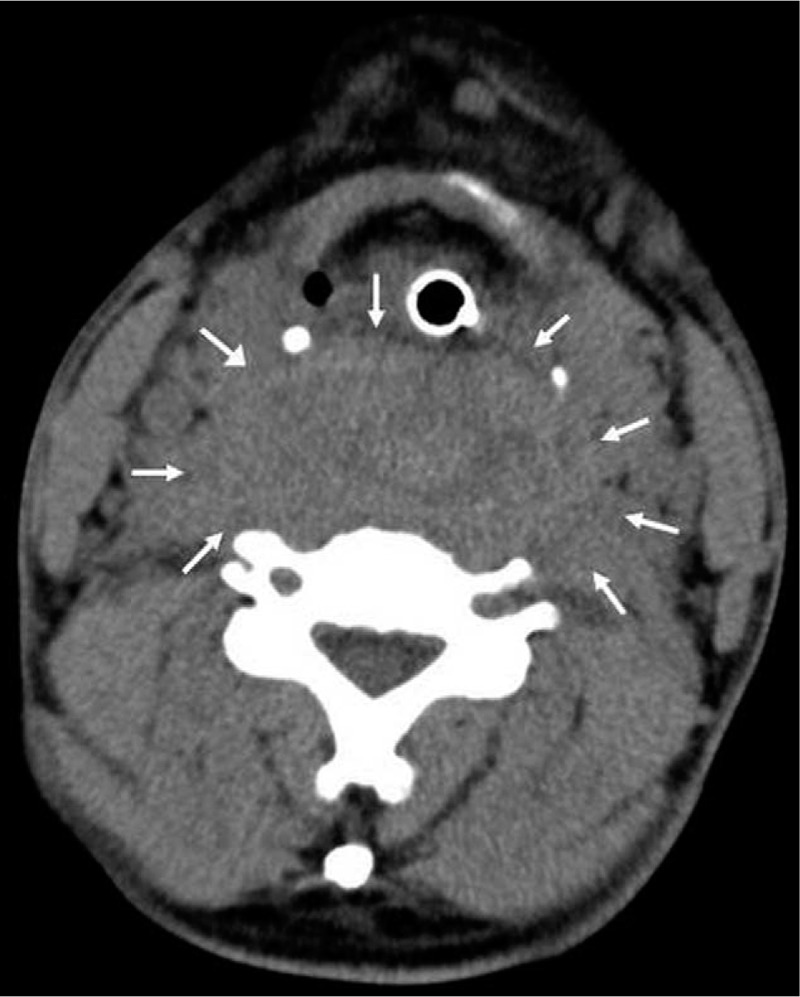
Post-intubation computed tomographic image of the neck. Axial view shows a retropharyngeal hematoma occupying the whole retropharyngeal space (arrows) and the endotracheal tube maintaining airway patency.

**Figure 3:**
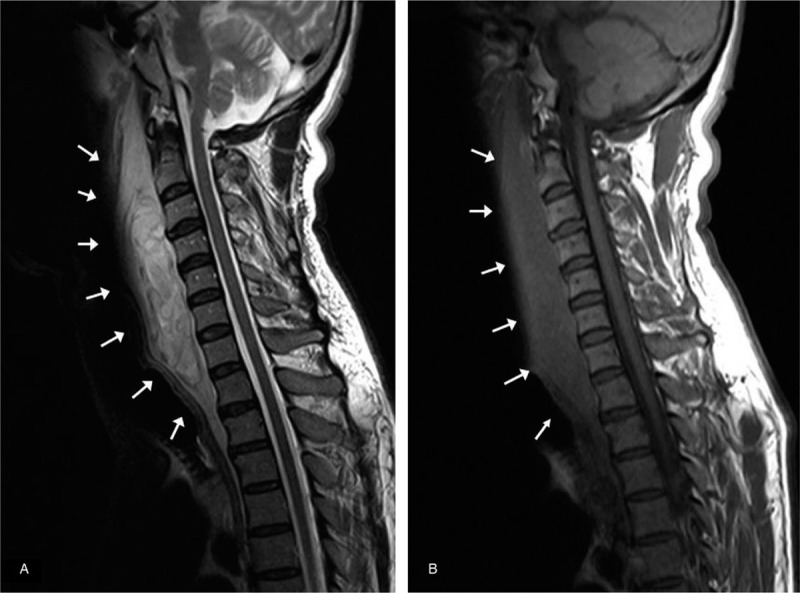
(a) T2-weighted sagittal Magnetic Resonance Imaging shows a longitudinal mass with heterogenous signal intensity in the retropharyngeal space (arrows). (b) T1-weighted sagittal Magnetic Resonance Imaging shows a mass with low signal intensity in the retropharyngeal space (arrows).

X-ray and neck computed tomography (CT) were performed. A lateral neck X-ray showed widening of the prevertebral space (Fig. 1), and neck CT with intravenous contrast revealed an intense retropharyngeal hematoma with no definite fracture or vessel injury (Fig. 2). MRI showed no definite fracture or ligament injury. A longitudinal and relatively well-defined mass with inhomogeneities in the retropharyngeal space from the nasopharyngeal level to superior mediastinal level was revealed on T2-weighted sagittal magnetic resonance imaging (MRI) (Fig. 3A). The longitudinal mass showed low signal intensity on T1-weighted sagittal MRI (Fig. 3B). We could not determine the possible cause, but we surmised the mass to be a huge retropharyngeal hematoma. Ear, nose, and throat (ENT) consultation was conducted.

Immediate neck exploration was done for the huge hematoma removal and bleeding control as the time for a huge hematoma to be absorbed is expected to be long, which further is considered risky due to possibility of infections such as deep neck infection, mediastinitis, or pneumonia. No vascular injury or active bleeding was identified and catheters for drainage were inserted. Ventilator weaning and extubation was performed on the 7^th^ postoperative day, and the patient was discharged without any postoperative sequelae on the 30^th^ postoperative day.

The Human Research Ethics committee of Yeungnam University Hospital waived the requirement of ethical approval, as this is a single case report. The patient provided written informed consent for publication of the clinical details and images pertinent to this case.

## 3 Discussion

The present case shows that a small amount of retropharyngeal hemorrhage secondary to minor trauma in young patients could be easily missed in the primary assessment. In the present case, a young patient with a light neck hyperextension injury without apparent soft tissue injury in a minor collision was discharged with a recommendation to revisit the emergency medical center upon onset of any symptoms. The patient developed a huge retropharyngeal hematoma within only 2 hours after hospital discharge. The imaging modality of choice is CT, but since it is not routinely performed in the emergency department, retropharyngeal hematomas due to minor injury ironically gain time to increase in size. Hence, another novel diagnostic strategy is essential to reduce the missed injury rate of this rare and potentially fatal pathology associated with minor trauma.

Retropharyngeal hematoma has various causes, including cervical trauma, neck surgery, deep neck infections, anticoagulant therapy, cannulation, foreign body ingestion, retropharyngeal infection, carotid artery aneurysm, even coughing or vomiting.^[1–3]^ Among these, retropharyngeal hematoma secondary to blunt trauma is extremely rare, only approximately 63 cases have been reported.^[2–3,9]^ In our interest, life-threatening large retropharyngeal hematoma develops more often in minor trauma than in high energy trauma.^[9]^ Particularly, minor trauma and hyperextension are usually associated with life-threatening large hematoma and delayed onset of symptoms.^[9–10]^

The low incidence rate and poor understanding of this pathology is another reason why this condition is easily undiagnosed. Anatomically, retropharyngeal space is a compact but potential space that is located between the pharynx and spine. It is usually impossible to discern the virtual space from the surrounding tissue in a normal patient in imaging studies.^[1]^ Fluid, such as water or blood, entering the retropharyngeal space emerges separated from the surrounding tissue, displacing the buccopharyngeal fascia anteriorly and prevertebral fascia posteriorly.^[2]^ Accurate knowledge of the normal anatomy and the imaging studies used to evaluate this potential space will help doctors who encounter them in prompt and correct diagnosis in emergency settings. Additionally, suspicion and routinely performed CT can be helpful.

Accurate and rapid diagnosis without missing the injury is essential for good prognosis of this pathology because the management is relatively simple. Initial treatment of retropharyngeal hematoma is to secure the airway.^[9–10]^ After that, the treatment of choice is conservative therapy with close observation, although occasional emergency surgery might be required.^[2–3,9–10]^ Most hematomas regress spontaneously in 2 to 4 weeks.^[2–3]^

High level of suspicion and a novel diagnostic strategy for close observation of the patient in the hospital for at least 24 hours are required to reduce the incidence rate of missed injuries. Researches to reduce the incidence rate of missed injuries and to identify the risk factors of missed injuries in initial survey have been performed actively.^[6,7,8,11]^ Risk factors associated with undetected injuries include young age, high Injury Severity Score, polytrauma, absence of soft tissue injuries, and presence of chest or pelvic injuries.^[12]^ Interestingly, young age was one of the significant risk factors of undetected injuries in emergency department in a retrospective cohort study.^[12]^

The newly developed tertiary survey refers to a novel protocol consisting of physical re-examination, history taking, and reviewing of imaging and blood results 24 hours after primary and secondary survey.^[7]^ Some studies demonstrate that this protocol is beneficial for the detection of missed injuries.^[7,11]^ One observational study reported that neglected lesions were observed in 11.5% patients in the protocol,^[11]^ and another literature review study reported missed injury detection rates in several studies to be better in the tertiary survey group, compared to primary and secondary survey groups, showing detection rates of missed injuries varying from 2.6% to 30.96%.^[7]^

In conclusion, this rare and life-threatening huge mass in the retropharyngeal space can be simply cured with only conservative treatment, if diagnosed early. High level of suspicion and re-examination of the patient after some time is essential to reduce the incidence rate of missed retropharyngeal hematoma and save precious lives. Tertiary survey performed 24 hours after primary survey and routinely checked CT can be a smart solution to prevent disastrous complications. Certainly, further research for minimizing missed injuries at primary survey should be recommended. A strategy for appropriate examinations including physical examination, history taking, imaging techniques, and laboratory tests should be developed for accurate and early diagnosis at primary assessment without missing injuries.

## Author contributions

**Conceptualization:** Jong Hyun Baek, Jung Hee Kim.

**Funding acquisition:** Jong Hyun Baek.

**Investigation:** Jong Hyun Baek, Jung Hee Kim.

**Supervision:** Jung Hee Kim.

**Validation:** Jong Hyun Baek, Jung Hee Kim.

**Visualization:** Jong Hyun Baek, Jung Hee Kim.

**Writing – ORIGINAL DRAFT:** Jung Hee Kim.

**Writing – review & editing:** Jong Hyun Baek, Jung Hee Kim.
